# Identification of a Novel Small Molecule STING Agonist Reshaping the Immunomicroenvironment of Pancreatic Ductal Adenocarcinoma

**DOI:** 10.7150/ijbs.107837

**Published:** 2025-05-27

**Authors:** Pengyi Liu, Minmin Shi, Yang Liu, Yihao Liu, Jiayu Lin, Shuyu Zhai, Yizhi Cao, Dongjie Chen, Yongsheng Jiang, Chenghong Peng, Lei Zhang, Chunyong Ding, Lingxi Jiang, Baiyong Shen

**Affiliations:** 1 Department of General Surgery, Pancreatic Disease Center, Ruijin Hospital, Shanghai Jiao Tong University School of Medicine, Shanghai 200025, China.; 2 Research Institute of Pancreatic Diseases, Shanghai Key Laboratory of Translational Research for Pancreatic Neoplasms, Shanghai Jiao Tong University School of Medicine, Shanghai 200025, China.; 3 State Key Laboratory of Oncogenes and Related Genes, Institute of Translational Medicine, Shanghai Jiao Tong University, Shanghai 200025, China.; 4 Ruijin Department of General Surgery, Ruijin Hospital, School of Medicine, Shanghai Jiaotong University, Shanghai, China; 5 Shanghai Frontiers Science Center for Drug Target Identification and Delivery, School of Pharmaceutical Sciences, Shanghai Jiao Tong University, Shanghai 200240, China.; 6 National Key Laboratory of Innovative Immunotherapy, Shanghai Jiao Tong University, 800 Dongchuan Road, Minhang District, Shanghai 200240, China.

**Keywords:** Pancreatic ductal adenocarcinoma, STING agonist, immune microenvironment, T cells, macrophage polarization, anti-PD-1 therapy.

## Abstract

**Background:** Pancreatic ductal adenocarcinoma (PDAC) is a highly aggressive malignancy with limited treatment options and poor response to immunotherapy. The immunosuppressive tumor microenvironment, characterized by a dense extracellular matrix, and immunosuppressive cells, plays a crucial role in this resistance. The cGAS-STING pathway, traditionally recognized for antiviral defense, has emerged as a potential target for cancer immunotherapy due to its ability to activate both innate and adaptive immune responses.

**Methods:** A novel small-molecule STING agonist, D166, was synthesized by incorporating deuterium into the structure, leading to improved stability and activation of the STING pathway. The effects of D166 were evaluated using human pancreatic tumor organoids, mouse pancreatic tumor models, and various *in vitro* and *in vivo* assays, including flow cytometry, RNA sequencing, ELISA and western blotting. And an organoid-immune cells co-culture system was established for further investigate the effects on immune cells.

**Results:** D166 demonstrated significant anti-tumor activity, effectively activating the cGAS-STING pathway in a time- and dose-dependent manner. D166 inhibited the progression of pancreatic tumor organoids and mouse pancreatic tumors, reshaping the tumor immune microenvironment. The drug enhanced T cell activation, promoted macrophage polarization toward the M1 phenotype, and increased the infiltration of immune cells. Additionally, D166 acted as a sensitizer for anti-PD-1 therapy, significantly improving therapeutic efficacy in combination treatments.

**Conclusion:** D166 is a novel and stable STING agonist that inhibits pancreatic tumor progression by activating the cGAS-STING pathway and remodeling the tumor immune microenvironment. Its combination with anti-PD-1 antibodies offers a promising strategy for overcoming the immunosuppressive barriers in pancreatic cancer, providing new therapeutic insights and directions.

## Introduction

Pancreatic ductal adenocarcinoma (PDAC) is an exceptionally aggressive malignancy, with a 5-year survival rate of <10%, and may become the second leading cause of cancer-related mortality in the near future 1. Most patients are diagnosed at the advanced stage due to the absence of early clinical signs. Currently, surgical resection with chemotherapy is the primary treatment approach. However, its overall efficacy is limited, highlighting the urgent need for novel and more effective therapeutic strategies.

Although immunotherapy has revolutionized cancer treatment, its effectiveness in solid tumors, particularly “cold tumors,” such as pancreatic cancer, remains limited 2,3. Established immunotherapies, including CAR-T cell, anti-PD-1, and anti-CTLA4 therapies, provide minimal benefit in PDAC, largely due to its profoundly immunosuppressive microenvironment. The PDAC microenvironment is rich in immunosuppressive components, including myeloid-derived suppressor cells, M2-polarized macrophages, regulatory T cells, pancreatic stellate cells, and inhibitory cytokines such as interleukin (IL)-10, transforming growth factor-β, and IDO. These factors render PDAC an “immunosuppressive disease” 4. Ongoing efforts aim to activate both innate and adaptive immunity within this hostile tumor microenvironment (TME) to improve therapeutic efficacy.

One emerging strategy leverages immunotherapy to stimulate type I interferons (IFNs), which orchestrate anti-tumor cytokine production and modulate innate immune responses upon binding to their receptor complex (IFNAR) on the cell surface 5. The stimulator of interferon genes (STING)—an endoplasmic reticulum-associated protein that triggers the phosphorylation and activation of the transcription factor IRF3—is the key regulator of this pathway 6,7. Once activated, IRF3 translocates to the nucleus and promotes the expression of pro-inflammatory genes essential for mounting an effective anti-tumor response.

The cyclic GMP-AMP synthase (cGAS)-STING pathway was initially identified as a cytosolic DNA sensor that primarily functions in antiviral defense 8. Various sources, including DNA damage, genomic instability, damaged mitochondria, exosomes, reverse transcription, DNA viruses, and bacteria, produce cytosolic double-stranded DNA. When cGAS binds to DNA, it undergoes a conformational change and catalyzes the synthesis of 2',3'-cyclic GMP-AMP (cGAMP) from ATP and GTP. cGAMP then acts as a secondary messenger, transmitting the signal to a downstream STING molecule in the endoplasmic reticulum. Upon activation, STING triggers a cascade via TBK1 and IRF3, inducing type I IFNs and nuclear factor-kappa B (NF-κB)^9^. Both IRF3 and NF-κB then translocate to the nucleus, enhancing type I IFN and IL-6 expression and activating innate and adaptive immune responses. Notably, the cGAS-STING pathway plays a crucial role in anti-tumor immunity 7,10,11.

Consequently, the development of STING agonists has attracted significant attention, and several studies have investigated their potential in cancer therapy. One such agonist is 5,6-Dimethylxanthenone-4-acetic acid (DMXAA), which modulates immune responses and exerts anticancer effects in preclinical models 12. However, it activates the cGAS-STING pathway only in mice, making it unsuitable for human cancer treatment. STING agonists that mimic cyclic dinucleotide (CDN) binding, such as ADU-S100, have entered clinical trials but exhibit poor stability 13. More stable synthetic small-molecule agonists, including diABZIs, MSA-2, and SR-717, have been identified using high-throughput screening 14-16. Additionally, STING agonists can also be used as cancer vaccines to stimulate immune responses and prevent tumor development. STING-dependent vaccines have demonstrated the potential to inhibit tumor growth and induce long-lasting anti-tumor immunity 17,18. Nevertheless, current STING agonists exhibit certain challenges. The low structural similarity between human and mouse STING has contributed to clinical trial failures 15. Other unresolved issues include drug instability, immune-related side effects, and challenges with drug delivery 19.

In this study, we introduced a novel small-molecule STING agonist, D166. As monotherapy, D166 effectively inhibited pancreatic cancer progression and reprogramed the tumor immune microenvironment. Furthermore, it activated T cells within pancreatic tumors, enhanced their cytotoxic function, and promoted macrophage polarization toward the pro-inflammatory M1 phenotype. In addition, in combination with anti-PD-1 therapy, D166 demonstrated significant anti-tumor efficacy, offering promising insights and therapeutic possibilities for pancreatic cancer treatment.

## Methods

### Extraction and Detection of Pancreatic Tumor Organoids

Pancreatic tumor organoids were derived from patients diagnosed with pancreatic ductal adenocarcinoma at Ruijin Hospital, affiliated with Shanghai Jiao Tong University School of Medicine 20. Inclusion criteria required a confirmed histopathological diagnosis of pancreatic ductal adenocarcinoma and the absence of prior neoadjuvant treatment. After obtaining informed consent from the patients, tumor tissues were excised during surgery and preserved in a tissue preservation solution (Absin). The tumor samples were promptly minced into small fragments in pre-chilled RPMI-1640 and digested for 20 minutes at 37°C using the Human Tumor Dissociation Kit (Miltenyi Biotec). The resulting single-cell suspension was filtered through a 100-µm mesh (Falcon) and seeded onto Matrigel (Corning). Following a 30-minute incubation at 37°C, OM12 (Omabio) complete organoid medium was added around the periphery of the Matrigel. The tissue-derived cells successfully grew in a three-dimensional format.

The viability of the organoids was assessed using CellTiter-Glo® 3D Cell Viability Assay (Promega) reagents. The assay solution was added to the organoids, followed by vigorous shaking for 5 minutes. The mixture was then incubated at room temperature in the dark for 25 minutes. Luminescence was subsequently measured using the BioTek Synergy LX multimode reader (Agilent). To determine the viability of the organoids, AO/PI staining was employed. Acridine orange (AO) binds to the nuclei of all cells, emitting green fluorescence, while propidium iodide (PI) penetrates the cell membrane to stain dead cells red. After 20 minutes of AO/PI staining, images were captured using the Castor S1 (Countstar), allowing for the differentiation between live and dead cells within the organoids.

### Cell Culture and Transfection

Pancreatic tumor cell lines were obtained from the Cell Bank of the Chinese Academy of Sciences (Shanghai, China) and authenticated by short tandem repeat (STR) profiling. KPC cell line was extracted from KPC mice PDAC tumor and cultured. All cells were cultured in RPMI-1640 or Dulbecco's Modified Eagle Medium (DMEM), supplemented with 10% fetal bovine serum (FBS, Gibco) and 1% penicillin-streptomycin (P/S, NCM). The mouse pancreatic cancer cell lines, Pan02 and KPC, were stably transfected with a luciferase-expressing virus. Lentivirus and Hilymax were added to the culture medium, and after 8 hours, the medium was replaced. The cells were subsequently selected with 2 μg/mL puromycin to generate the Pan02-luc and KPC-luc cell lines. All cells and organoids were cultured at 37°C in a 5% CO2 environment.

### Co-culture of Organoids and Immune Cells

T cells and monocytes were isolated from the peripheral blood of patients using the EasySep™ Human CD8+ T Cell Isolation Kit and Human Monocyte Isolation Kit (STEMCELL). CD8+ T cells were activated with anti-CD3 (5 μg/mL) and anti-CD28 (5 μg/mL) antibodies (BioLegend), while monocytes were stimulated with 50 ng/mL of M-CSF (BioLegend) for 6 days to induce differentiation into macrophages. The activated T cells were subsequently added to pancreatic tumor organoid cultures pre-treated with the drug D166, or tumor organoids were co-cultured with monocyte-derived macrophages, forming a co-culture system. On day 5, immune cells were harvested for flow cytometry analysis. Tumor organoids were photographed, viability was assessed, and organoids were embedded for HE and IHC staining at days 0, 5, and 10 of co-culture.

### Mouse Tumor Model and Drug Application

C57BL/6 mice were obtained from Phenotek (Shanghai) and randomly assigned to groups, with a minimum of six mice per group. For the subcutaneous tumor model, approximately 5 × 10^5 tumor cells were suspended in 150 μL PBS and injected into the left flank of the mice. Tumor size was measured every four days starting from day 7 post-injection. On days 9, 12, and 15, the mice were administered three different concentrations of the drug via peritumoral injection, while the Control group received only the solvent. The mice were euthanized on day 25, and tumors were excised, weighed, and further analyzed.

For the pancreatic orthotopic tumor model, mice were anesthetized using alpha-chloralose (2,2,2-tribromoethanol) (Aladdin), and the pancreas was surgically exposed. Approximately 5 × 10^5 tumor cells suspended in 50 μL PBS were injected into the pancreas. The incision was then closed, and the mice were allowed to recover under close observation. On days 9, 12, and 15, the mice received intraperitoneal injections of three different concentrations of the drug. As with the subcutaneous model, the mice were euthanized on day 25, tumors were collected, weighed, and further analyzed. Additionally, at least 10 mice from each group were randomly selected to undergo the same treatments without euthanasia, and their survival times were recorded to generate survival curves.

For genetically engineered KPC mice [C57BL/6-Krastm1(LSL-G12D)Trp53tm1(LSL-R172H)Tg(Pdx1-Cre)], obtained from Cyagen (Shanghai), the mice were randomly divided into two groups. Beginning in the 6th week, the treatment group received weekly intraperitoneal injections of anti-PD-L1 antibody (100 μg, BioXCell) and D166 (10 mg/kg), while the Control group was given 100 μg IgG and the corresponding solvent. This treatment was administered once a week for a total of six weeks. Three mice were randomly euthanized at weeks 10, 12, and 14 for tumor size measurement. The remaining mice were used to monitor survival, and survival differences between the groups were analyzed statistically.

### *In vivo* Imaging in Mice

Pancreatic orthotopic tumor models were established using mouse pancreatic tumor cell lines (Pan02-luc and KPC-luc). Mice were intraperitoneally injected with 200 µL of D-Luciferin working solution (15 mg/mL) (Yeasen) and continuously anesthetized with isoflurane. Ten minutes after injection, the intensity and distribution of luciferase luminescence were measured using the Tanon ABL-X5 *in vivo* imaging system. Tumor size was monitored weekly, with consistent exposure times and intensities for imaging.

### RNA-seq and scRNA-seq

Bulk RNA sequencing was performed by GenePlus (China) on pancreatic cancer organoids treated with 10 µM of D166 or MSA-2, compared to a control group, with three biological replicates. The sequencing data are available for access and analysis via the provided website. Single-cell RNA sequencing (scRNA-seq) was conducted by NovelBio (China) on in situ mouse pancreatic tumors following tissue digestion. The resulting sequencing data were analyzed using R software.

### Western Blot

Tumor tissues or cells were thoroughly lysed in RIPA buffer containing protease and phosphatase inhibitors. The lysates were then centrifuged to collect the supernatant. Proteins were separated by electrophoresis in MOPS buffer and subsequently transferred onto a PVDF membrane. After blocking, the membrane was sequentially incubated with the primary antibody, secondary antibody, and chemiluminescent substrate (ECL) for imaging. Antibodies used in this article were listed in Table S1.

### Real-time Fluorescence Quantitative PCR (RT-qPCR)

RNA from tumor tissues or cells was extracted using the SteadyPure Universal RNA Extraction Kit (Accurate Biology) and reverse transcribed into cDNA with the Evo M-MLV Reverse Transcription Kit (Accurate Biology). Relative RNA expression levels were quantified using 2X Universal SYBR Green Fast qPCR Mix (Abclonal), and the results were recorded on the qTOWER®3 84 Real-Time PCR system. Primers used in this article were listed in Table S2.

### Cytokine Assay and ELISA

Multiplex cytokine detection was performed using Luminex cytokine bead array technology provided by WayenBio, Shanghai. In the mouse tumor model, levels of multiple cytokines were simultaneously measured and normalized to tumor weight. For the quantification of specific cytokines, such as IL-6 and IFN-β, enzyme-linked immunosorbent assay (ELISA) kits from Abclonal were used. The assays were conducted according to the manufacturer's instructions. ELISA kits used in this article were listed in Table S3.

### Hematoxylin and eosin (HE), immunohistochemistry (IHC), and immunofluorescence (IF) staining

After the mice were euthanized, tumor tissues were preserved in 4% formalin, paraffin-embedded, and sectioned. Hematoxylin and eosin (HE) staining was performed following standard protocols, and images were captured using a microscope. For immunohistochemistry (IHC) staining, the streptavidin-biotin-peroxidase complex method was used. The procedure included dewaxing, hydration, antigen retrieval, blocking, primary and secondary antibody incubation, DAPI staining, and mounting. For immunofluorescence (IF) staining, fluorescently labeled secondary antibodies were employed. Images were captured and processed using a confocal microscope (Zeiss).

### Flow Cytometry Analysis

Mouse tumors were digested using the Mouse Tumor Dissociation Kit (Miltenyi Biotec) according to the manufacturer's protocol. The resulting single-cell suspension was filtered through a 70-micron mesh to ensure a homogeneous cell population. Cells were stained with fluorescently labeled antibodies for flow cytometry at 4°C on ice for 30 minutes. After washing off unbound antibodies, fluorescence was detected using a Beckman CytoFlex S flow cytometer. For co-cultured immune cells, separated T cells and macrophages were similarly stained with flow cytometry antibodies. For cytokine staining, cells were pre-stimulated with the eBioscience™ Cell Stimulation Cocktail for 4 hours. For intracellular protein staining, after surface staining, cells were fixed and permeabilized using the BD Cytofix/Cytoperm™ Fixation/Permeabilization Kit, followed by staining for intracellular proteins. Flow cytometry data were analyzed using FlowJo 10 software. Antibodies used were listed in Table S1.

### Data Statistics

All experiments were performed in triplicate or more and results are presented as mean ± standard error of the mean (SEM). Statistical significance of tumor weight and cytokine levels were determined using Student's t-test, while survival differences in Kaplan-Meier curves were analyzed using the log-rank test, calculating the statistical differences between each treatment group and the control group. Data processing and analysis were conducted using GraphPad Prism software (Version 9.0). *p < 0.05; **p < 0.01; ***p < 0.001; ****p < 0.0001.

## Results

### Novel STING agonist synthesis

**Figure 1:**
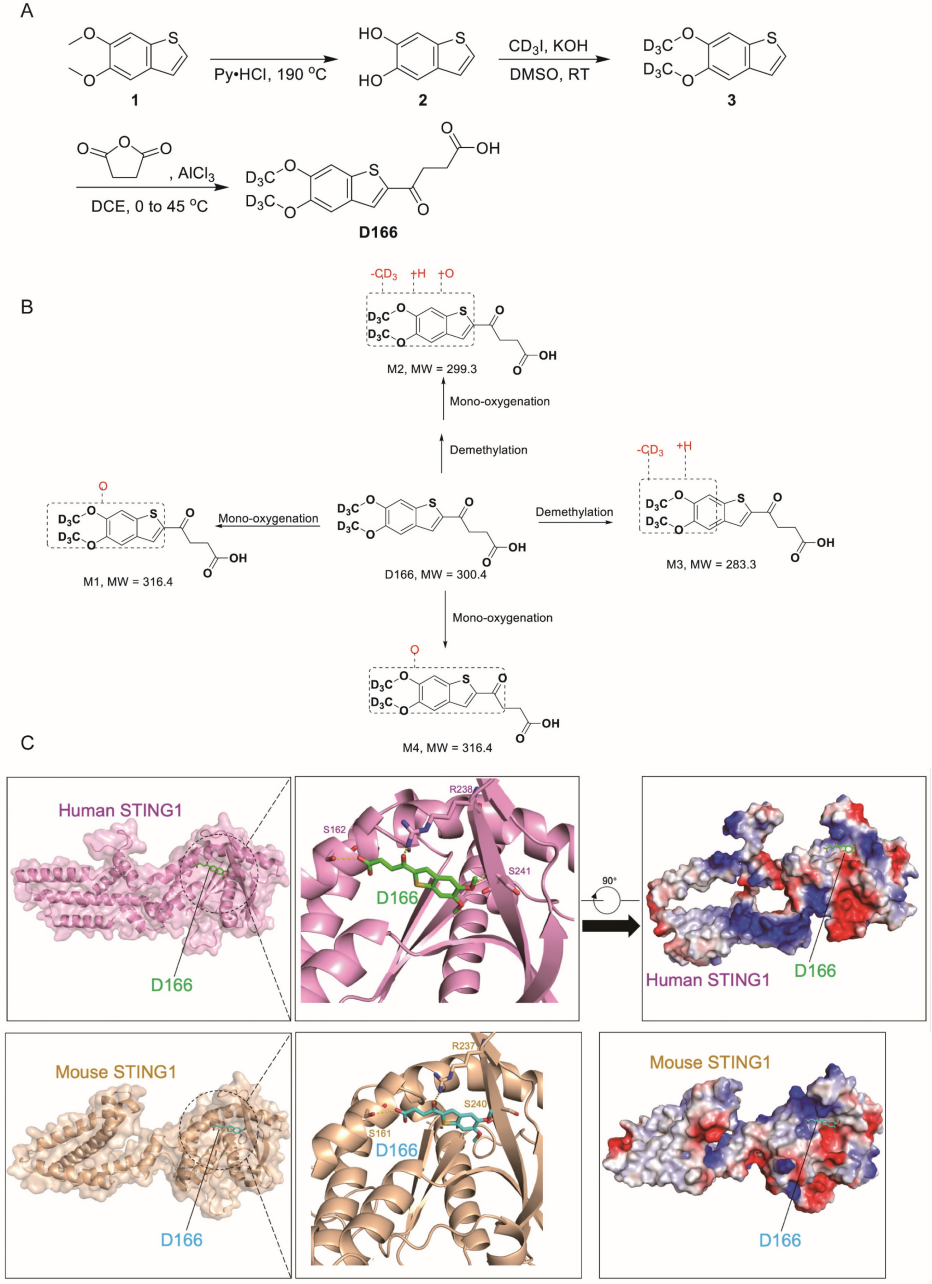
** Chemical synthesis of D166. (A)** The procedure of D166 synthesis. Benzo[b]thiophene-5,6-diol (2) was synthesized by compound 1 and pyridine hydrochloride. 5,6-bis(methoxy-d3)benzo[b]thiophene (3) was synthesized by compound 2, deuterated iodomethane and potassium hydroxide. D166 was synthesized by succinic anhydride, 1,2-dichloroethane and aluminium chloride. **(B)** Proposed metabolic pathway of D166 in mice, rats, dogs, and humans. **(C)** Molecular docking of D166 and human STING (above) and mouse STING (below). S for serine, R for arginine.

A novel STING agonist D166 was synthesized by enhancing the stability of the MSA-2 structure by incorporating deuterium (D) (Figure 1A). Detailed synthesis procedures are provided in Supplementary Methods (Chemical Synthesis of D166). Compared with MSA-2^15^, D166 exhibited superior activation of STING and its downstream signaling pathways in both mouse and human systems, with improved stability (Tables 1 and 2). D166 also exhibited thermal stability similar to that of ADU-S100 (Figure S1A, B)^13^. Figure 1B presents the potential metabolic products of D166 in mice, rats, dogs, and humans. Our results revealed almost no additional metabolic products after 1 h of detection, demonstrating the high stability of D166 (Table 3).

Molecular docking revealed that D166 could effectively bind to both human and mouse STING molecules, activating the cGAS-STING pathway (Figure 1C). In human STING1, the key interacting amino acids were S162, R238, and S241. The D166 molecule inserted into a pocket structure resembling a “pyramid,” formed by α7, α8, α9, α10, α11, β1, β4, and β6. The diagram is rotated 90° clockwise and presented with a charge distribution map. Although the primary amino acid sequences of mouse and human STING1 differ, the key amino acids at the critical binding sites are relatively conserved, with the D166 binding sites being nearly identical. Moreover, the three-dimensional structures of the two proteins are highly similar. D166 was located in a pocket region with a charge gradient, where the upper and lower parts were enriched in positive charges (facing the carbonyl and benzene ring planes) and negative charges (facing the sulfur atom in the five-membered ring), respectively, further stabilizing the binding conformation.

### D166 inhibits the progression of pancreatic cancer organoids

**Figure 2:**
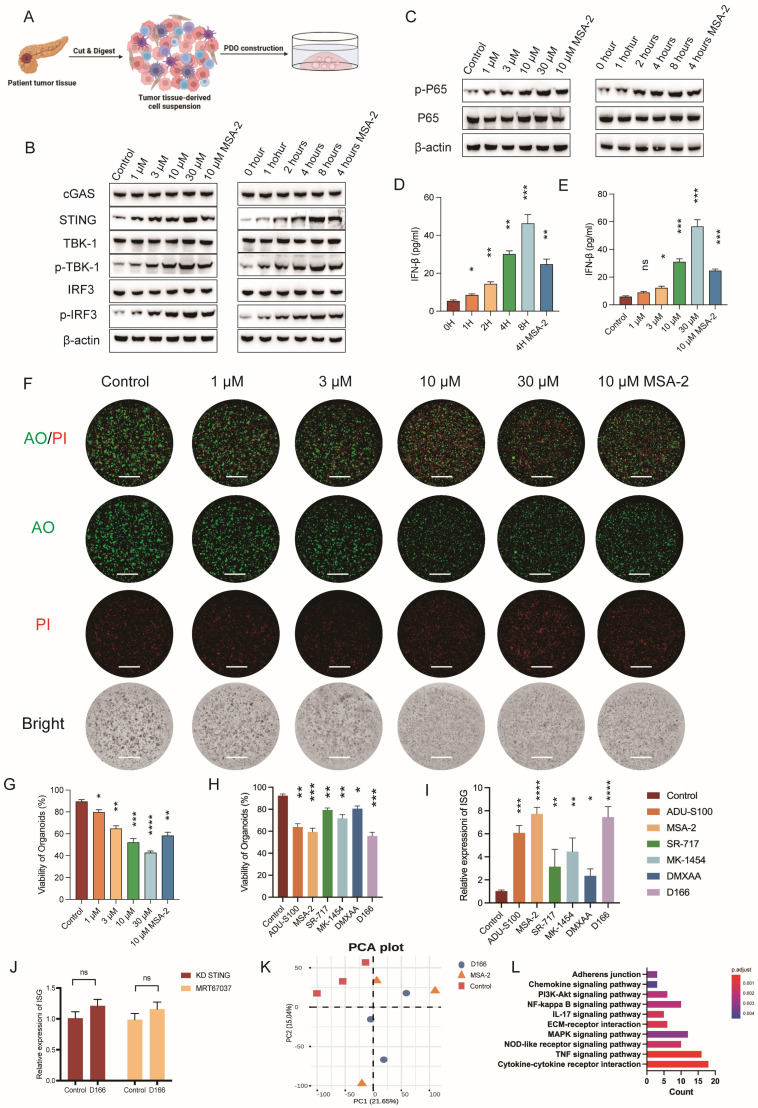
** D166 inhibits the progression of pancreatic cancer organoids. (A)** Schematic diagram of pancreatic cancer organoid construction. **(B-C)** Western blot analysis showing protein expression level after different concentration and duration of D166 adding to pancreatic cancer organoid. **(D-E)** The concentration of IFN-β in culture medium after different concentration (D) and duration (E) of D166 adding to organoids. **(F)** Representative picture of pancreatic cancer organoids after D166 treatment and AO/PI staining. AO represent for acridine orange, and PI represent for propidium iodide. Scale bar = 1 mm. **(G)** Percentage of organoid viability in (F). **(H)** Percentage of organoid viability after treatment by 10μM different STING agonists and AO/PI staining. **(I)** Relative ISG expression in pancreatic cancer organoids after treatment by 10μM different STING agonists. **(J)** Relative ISG expression of ISG gene after STING knockdown or adding MRT67037 to culture medium, normalized to control group. **(K)** PCA plot of bulk RNA sequencing of control, D166 and MSA-2 groups. **(L)** GESA pathway enrichment in D166 group compared with control group. Statistical differences between each treatment group and the control group were calculated using two-tailed unpaired Student's t-tests. *p < 0.05; **p < 0.01; ***p < 0.001; ****p < 0.0001.

We developed pancreatic tumor organoid models using tissues from patients with pancreatic cancer to enhance the clinical relevance and explore the therapeutic effects of D166 on pancreatic tumors (Figure 2A). D166 demonstrated a time- and dose-dependent effect in activating the cGAS-STING pathway and inducing IFN-β in pancreatic cancer organoids (Figure 2B-E). Furthermore, D166 also activated STING and downstream signaling molecules in human pancreatic cell line in a dose- and time-dependent manner without affecting proliferation (Figure S2A-D). AO/PI staining to differentiate live from dead cells revealed increased concentration of D166 and decreased viability of pancreatic tumor organoids, along with a corresponding reduction in volume (Figure 2F, G). Compared with previously reported STING agonists, including ADU-S100, MSA-2, SR-717, MK-1454 and DXMAA 12,16,21-23, D166 exhibited better efficacy in activating IFN-stimulated genes (ISGs) and inhibiting organoid viability (Figure 2H, I, Figure S2F, G). Next, we knocked down STING expression in pancreatic cancer organoids and added the TBK1 inhibitor MRT67037 to confirm whether D166 exerts its tumor-killing effects through the cGAS-STING pathway. Both interventions rescued the D166-induced ISG activation in organoids (Figure 2J). Additionally, RNA-seq demonstrated that the effects of D166 on pancreatic cancer organoids were similar to those of MSA-2 compared with the control group (Figure 2K, Figure S2E). Gene set enrichment analysis (GSEA) identified that D166 primarily activated inflammatory-related pathways, including the cytokine signaling, NF-κB, and tumor necrosis factor (TNF) signaling pathways (Figure 2L). Therefore, D166 provides superior activation of the cGAS-STING pathway in pancreatic ductal adenocarcinoma organoids compared with previously reported STING agonists.

### D166 monotherapy inhibits pancreatic tumor progression in mice

**Figure 3:**
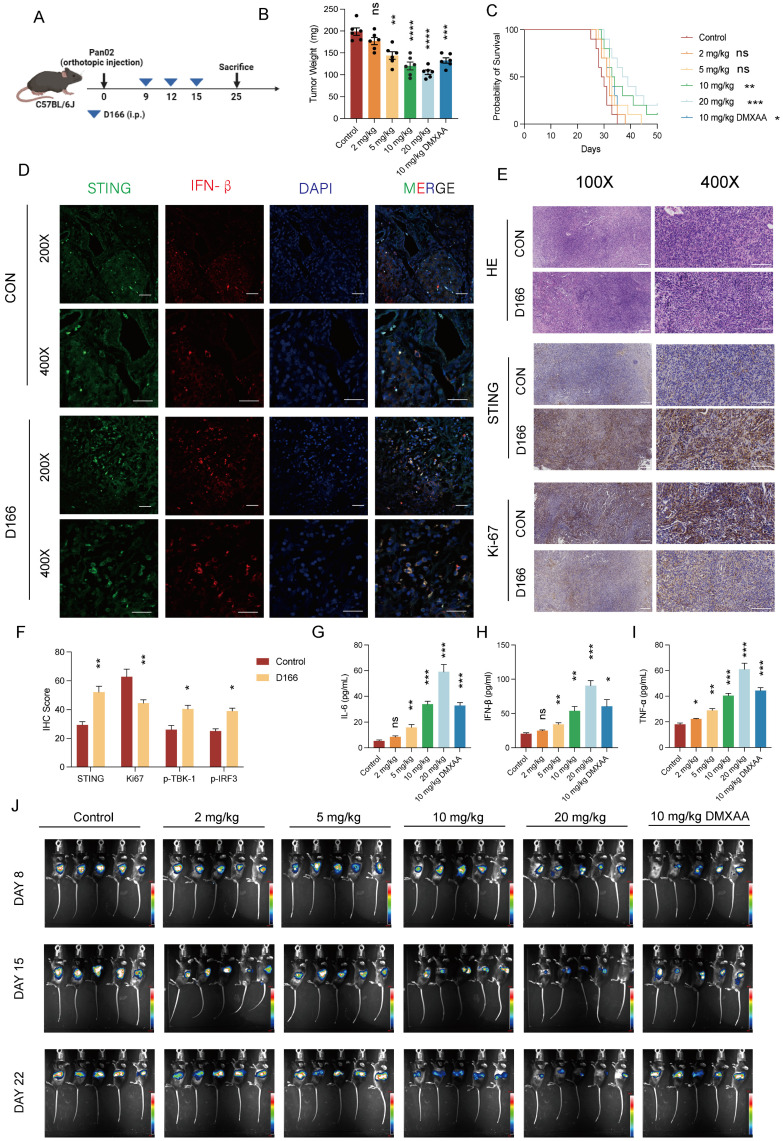
** D166 monotherapy inhibits pancreatic orthotopic tumor progression. (A)** Schematic diagram of mice pancreatic orthotopic tumor and D166 treatment. **(B)** Weight of mice pancreatic orthotopic tumor after sacrificed at day25, treated by different dose of D166 (male, n=6). **(C)** Survival curve of mice with orthotopic pancreatic cancer treated by different dose of D166 (n=10). **(D-E)** Representative IF (D) and IHC (E) image of orthotopic pancreatic cancer. Scale bar = 50 μm. **(F)** Relative IHC score of IHC images above. **(G-I)** The concentration of IL-6 (G), IFN-β (H) and TNF-α (I) in tumor microenvironment measured by ELISA. **(J)** *In vivo* imaging in mice after intraperitoneally injecting 15 mg/ml D-Luciferin. Tumor weight and cytokine levels were compared using two-tailed unpaired Student's t-tests, while survival differences in Kaplan-Meier curves were analyzed using the log-rank test, calculating the statistical differences between each treatment group and the control group. *p < 0.05; **p < 0.01; ***p < 0.001; ****p < 0.0001.

We developed a pancreatic tumor model using C57BL/6 mice to assess the *in vivo* effects of D166 (Figure 3A). Intraperitoneal injection of D166 at varying doses revealed a significant dose-dependent inhibition of pancreatic tumor progression (Figure 3B, C). Western blot, Immunofluorescence (IF) and immunohistochemistry (IHC) staining further demonstrated significant upregulation of STING pathway expression and reduced tumor proliferation following D166 treatment (Figure 3D-F, Figure S3A). Furthermore, higher D166 doses markedly increased STING pathway protein expression and elevated cytokine levels in the TME, including IL-6, IFN-β, and TNF-α (Figure 3G-I, Figure S3A). Compared with the traditional murine STING agonists, D166 treatment not only inhibited tumor proliferation but also promoted the release of IFN-β (Figure S3B, C). Weekly *in vivo* imaging showed that increasing D166 concentrations significantly inhibited tumor proliferation, demonstrating a more potent effect than DMXAA (Figure 3J, Figure S3D).

**Figure 4:**
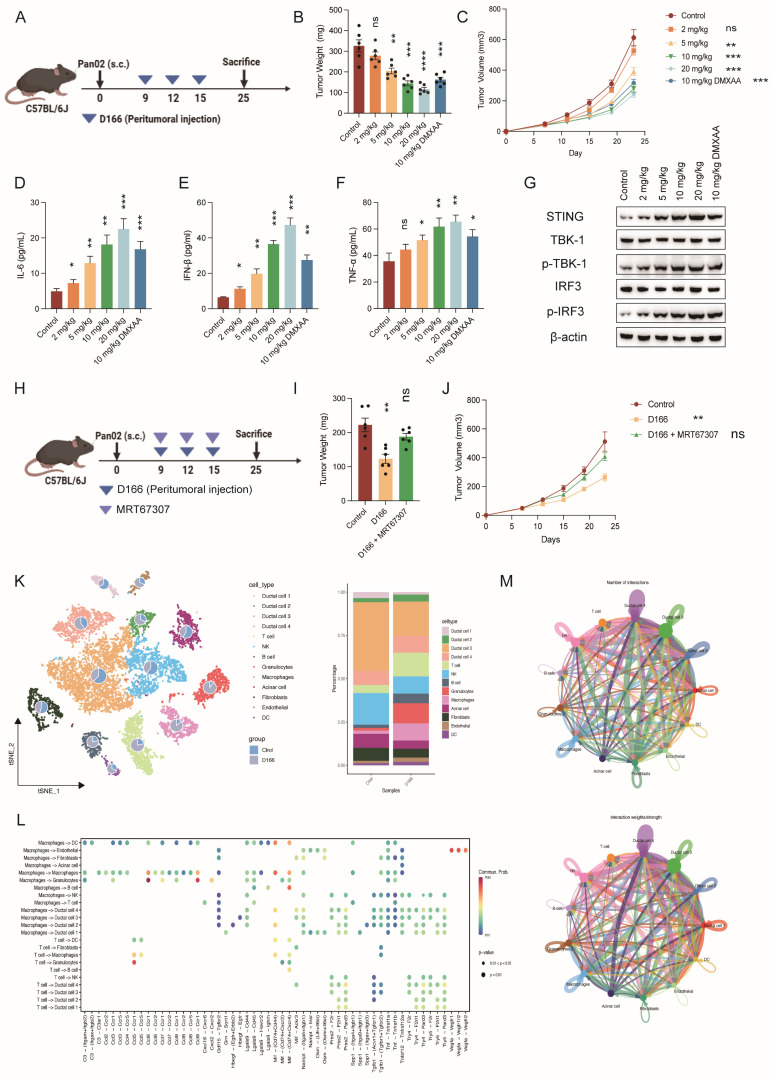
** D166 inhibits pancreatic cancer by altering cellular components. (A)** Schematic diagram of mice subcutaneous pancreatic tumor model and D166 treatment. **(B-C)** Tumor weight (B) and growth curve (C) of mice subcutaneous pancreatic tumor, treated by different dose of D166 (male, n=6). **(D-F)** The concentration of IL-6 (D), IFN-β (E) and TNF-α (F) in tumor microenvironment measured by ELISA. **(G)** Protein expression of STING and downstream key molecular in mice tumors by western blotting. **(H)** Schematic diagram of mice subcutaneous pancreatic tumor model and drug treatment. **(I-J)** Tumor weight (I) and growth curve (J) of mice subcutaneous pancreatic tumor, treated by different dose of drugs (n=6). **(K)** T-SNE map and cell proportion in control and D166 treatment group by single-cell RNA sequencing (n=3). **(L-M)** Cell-cell communication and cytokines analysis in pancreatic cancer microenvironment. Top: control group; Bottom: D166 treatment group. Tumor weight and cytokine levels were compared using two-tailed unpaired Student's t-tests, calculating the statistical differences between each treatment group and the control group. *p < 0.05; **p < 0.01; ***p < 0.001; ****p < 0.0001.

Subsequently, we established a subcutaneous pancreatic tumor model (Figure 4A). Peritumoral injection of D166 exhibited a significant dose-dependent inhibitory effect on tumor growth (Figure 4B-C). Moreover, the antitumor efficacy of D166 was comparable to that of other STING agonists (Figure S4A). IF and IHC staining indicated increased STING expression and phosphorylation of its downstream molecules, TBK1 and IRF3 (Figure S4B-E). Furthermore, higher D166 doses increased IL-6, IFN-β, and TNF-α levels in the TME (Figure 4D-F). Western blotting confirmed a progressive increase in STING expression and phosphorylation levels of its downstream targets, TBK1 and IRF3, with D166 treatment (Figure 4G). HE staining revealed reduced density of pancreatic tumors following D166 treatment. Pancreatic cancer exhibits a high tumor density, which majorly contributes to its immunotherapy resistance. However, the subcutaneous tumors appeared macroscopically softer following D166 administration, which was also observed in the HE sections (Figure S4E). These findings suggest that D166 may enhance the efficacy of immunotherapies, such as anti-PD-1 therapy, against pancreatic tumors.

Additionally, we co-administered the TBK1 phosphorylation inhibitor, MRT67037, with D166. MRT67037 counteracted the tumor-killing effect of D166 by blocking the D166-induced activation of the STING pathway (Figure 4H-J). Taken together, D166 inhibited pancreatic tumor progression by activating the STING pathway in a mouse model, providing a theoretical basis for combining D166 with immunotherapies.

### D166 reshapes the tumor immune microenvironment in pancreatic cancer

We performed 10X single-cell sequencing of the pancreatic orthotopic tumors to better understand how D166 exerts its inhibitory effects on pancreatic tumor progression and its target cellular components (Figure S3B). A significant reduction in the proportion of ductal cells (including cancer cells) and fibroblasts was observed following treatment with 10 mg/kg D166. This correlates with the HE results, where tumors in the D166-treated group appeared less dense, suggesting a transition from “cold tumors” to “hot tumors.” In contrast, the proportions of various immune cell types, particularly T cells, B cells, granulocytes, and macrophages, increased to varying degrees (Figure 4K). Additionally, cell-cytokine communication and cell-cell interaction analyses revealed relatively close interactions of T cells, NK cells, and macrophages with ductal cells (Figure 4L, M). Therefore, D166 inhibits pancreatic tumor progression by activating both innate and adaptive immunity, effectively reshaping the immune microenvironment within pancreatic tumors.

**Figure 5:**
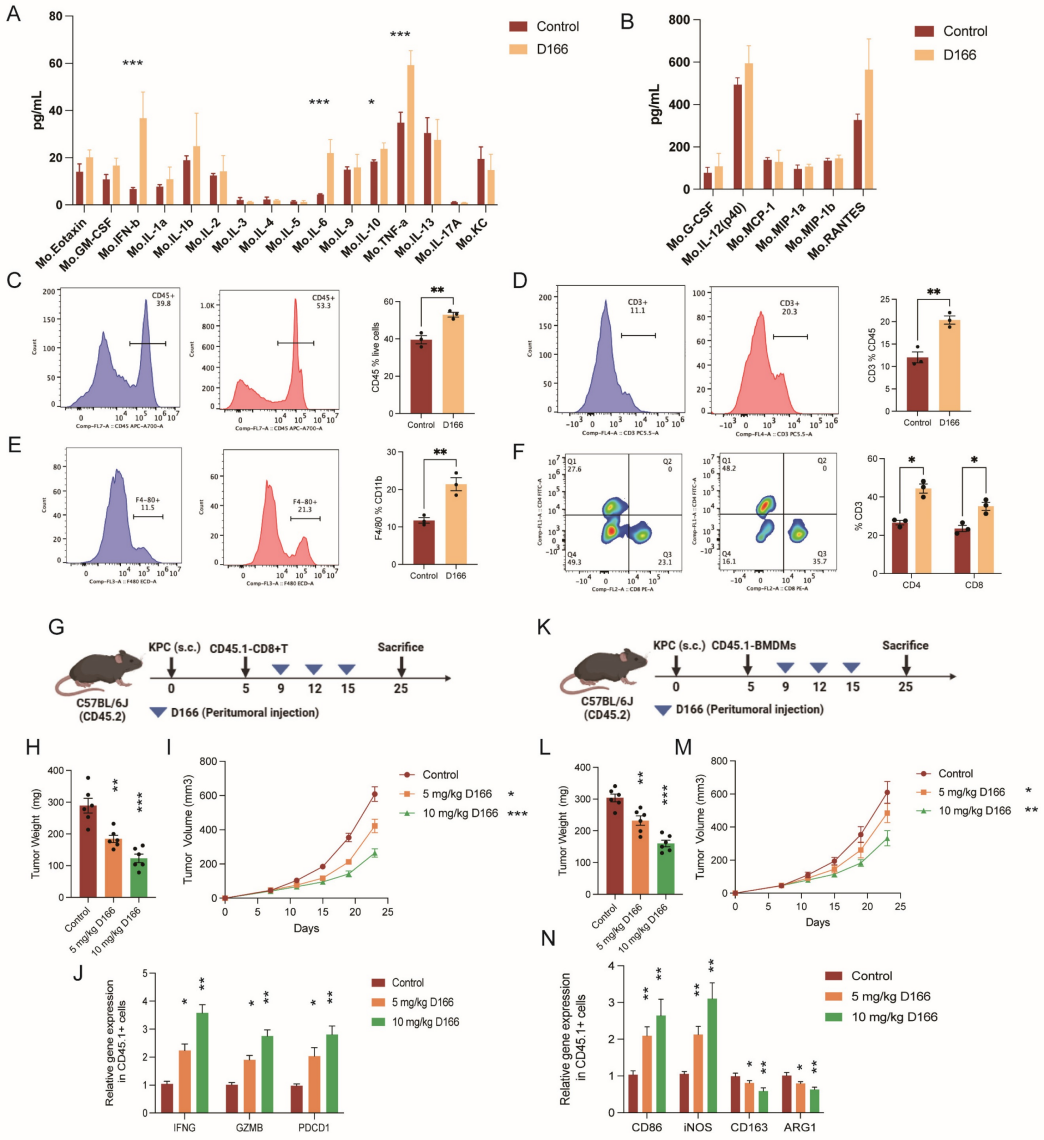
** D166 reshapes the tumor immune microenvironment in pancreatic cancer. (A-B)** Cytokines detection in mice pancreatic orthotopic tumors by Luminex cytokine bead array technology (n=3). **(C-F)** Flow analysis of the expression of CD45 (C), CD3 (D), F4/80 (E), CD4 and CD8 (F) in mice orthotopic PDAC microenvironment after 10 mg/Kg D166 treatment. The control group is shown on the left, the D166-treated group in the middle, and the statistical analysis on the right (n=3). **(G)** Schematic diagram of CD45.1+ T cells transferred to CD45.2 mice before D166 treatment. **(H-I)** Tumor weight (H) and growth curve (I) of subcutaneous pancreatic tumor, treated by different dose of D166 (n=6). (J) Relative gene expression in transferred CD45.1+ T cells. **(K)** Schematic diagram of CD45.1+ BMDM transferred to CD45.2 mice before D166 treatment. **(L-M)** Tumor weight (L) and growth curve (M) of subcutaneous pancreatic tumor, treated by different dose of D166 (n=6). **(N)** Relative gene expression in transferred CD45.1+ macrophages. Statistical differences between each treatment group and the control group were calculated using two-tailed unpaired Student's t-tests. *p < 0.05; **p < 0.01; ***p < 0.001; ****p < 0.0001.

Crosstalk between tumor and immune cells occurs primarily through cytokine-mediated signaling pathways 24. Multi-cytokine detection revealed increased levels of various cytokines following D166 treatment, with the most significant increases in IL-6, IFN-β, and TNF-α levels, aligning with previous studies (Figure 5A, B). Flow cytometry analysis of single-cell tumor suspensions revealed shifts in cell-type proportions within the pancreatic cancer microenvironment (Figure 5C-F, Figure S5A-D). Additionally, immune cell populations, particularly T cells and CD11b positive myeloid cells, were markedly increased. Among the T cells, we identified a CD3+CD4˗CD8˗ subset characterized by TCRγδ+ expression, indicating γδT cells (Figure S5E). D166 treatment significantly increased the proportion of CD4+ and CD8+ T cells. Moreover, a significant increase in F4/80-positive macrophages was observed among the myeloid cells. Therefore, D166 can activate T cells and promotes macrophage proliferation in PDAC microenvironment.

Next, we extracted T cells from CD45.1 mice, transferred them into CD45.2 tumor-bearing mice, and treated the recipients with D166 (Figure 5G). Tumor progression slowed as the D166 concentration increased (Figure 5H, I). Interestingly, sorting of CD45.1 cells revealed significant upregulation of IFNG, GZMB, and PDCD1 in the CD45.1 T cells (Figure 5J). Similarly, when bone marrow-derived macrophages from CD45.1 mice were transferred into CD45.2 tumor-bearing mice, D166 treatment increased the expression of M1 macrophage markers (CD86 and iNOS) in CD45.1 macrophages, whereas the expression of M2 markers decreased (Figure 5K-N). These results indicate that D166 enhances T cell activation and cytotoxicity, even in exogenous immune cells, and promotes M1 polarization of macrophages.

### D166 activates T cells and influences macrophage polarization

**Figure 6:**
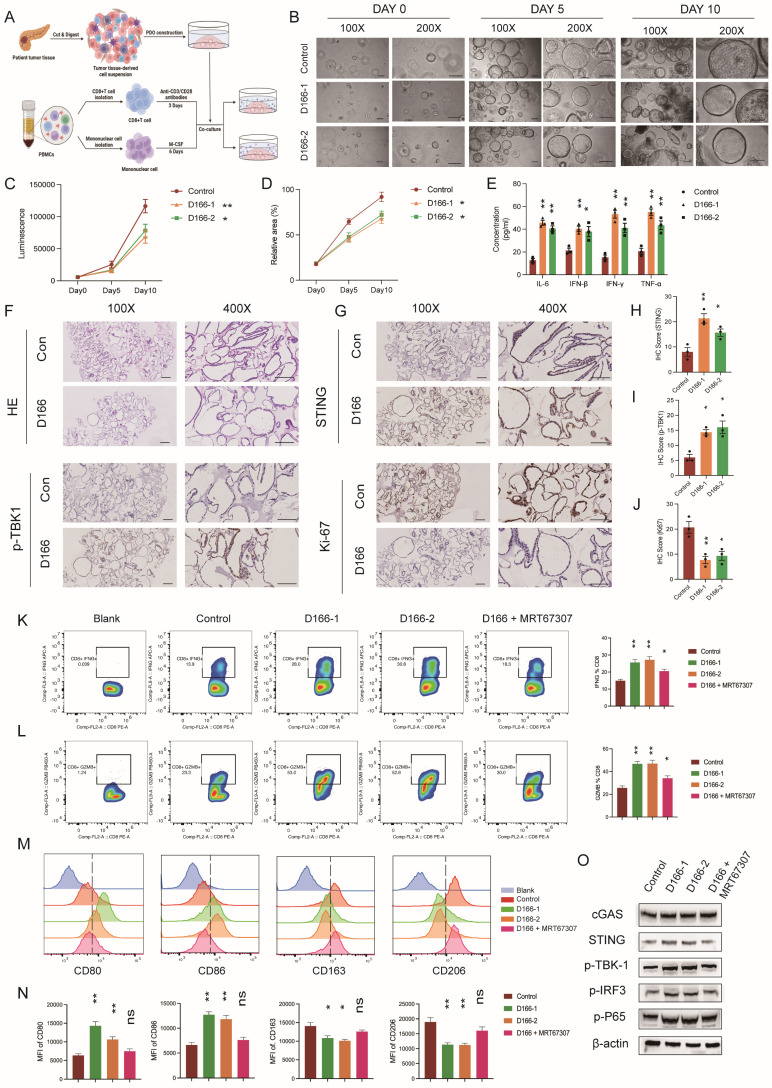
** D166 activates T cells and impact on macrophage polarization. (A)** Schematic diagram of pancreatic cancer organoids co-cultured with immune cells after treated by D166. **(B-D)** Representative images (B), luminescence by CTG (C) and relative area of organoids (D) of co-cultured organoids in day0, day5 and day10. **(E)** The concentration of IL-6, IFN-β and TNF-α in cell culture medium measured by ELISA. **(F-J)** HE staining (F), IHC staining (G) and relative IHC score (H-J) of pancreatic cancer organoids. Scale bar = 50 μm. **(K-L)** The expression of IFNG (K) and GZMB (B) in co-cultured T cells analyzed by flow after different treatments on organoids. The representative flow cytometry plots are shown on the left, and the statistical analysis is shown on the right. **(M-N)** Representative flow image (M) and statistical analysis (N) of co-cultured macrophages analyzed by flow cytometry after different treatments on organoids. **(O)** Protein expression of STING pathway in organoids after different treatments. Statistical differences between each treatment group and the control group were calculated using two-tailed unpaired Student's t-tests. *p < 0.05; **p < 0.01; ***p < 0.001; ****p < 0.0001.

We established an *in vitro* co-culture model of pancreatic cancer organoids and immune cells to further validate the effects of D166 on T cells and macrophages. Human peripheral blood-derived T cells were sorted and activated with CD3/CD28 antibodies, whereas human peripheral blood-derived monocytes were sorted and activated with M-CSF, followed by co-culturing with pancreatic tumor organoids (Figure 6A). Organoid viability was assessed on days 0, 5, and 10, and immune cell function was analyzed using flow cytometry. The CTG assay revealed a significant reduction in size and viability of pancreatic cancer organoids following D166 treatment (Figure 6B-D). Cytokine levels were measured using ELISA. Consistent with the *in vivo* findings, IL-6, IFN-β, IFN-γ, and TNF-α levels were significantly increased (Figure 6E). Similarly, IHC results indicated that D166 activated the STING pathway in pancreatic cancer organoids, suppressing organoid proliferation (Figure 6F-J).

Flow cytometry analysis revealed a marked increase in IFNG and GZMB release from T cells, along with the upregulation of Ki67 and PD-1 expression (Figure 6K-L, Figure S6A-B). Consistent with *in vivo* flow analysis (Figure S5D), these results suggest a potential benefit when combined with anti-PD1 treatment. In macrophages, the mean fluorescence intensities (MFI) of CD86 and CD80 (M1 markers) were significantly increased, whereas those of CD163 and CD206 (M2 markers) were significantly decreased (Figure 6M, N). It is noteworthy that these immune cell alterations were tightly linked to STING pathway activation within the organoids (Figure 6O). Following the addition of a TBK1 inhibitor, the modulatory effects of D166 on both T cells and macrophages were significantly diminished or even completely abrogated. Therefore, D166 enhances the secretion of pro-inflammatory cytokines from pancreatic cancer organoids; promotes T cell activation, proliferation, and cytotoxic activity; and induces a shift in macrophages towards the M1 anti-tumor phenotype through cytokine signaling, thereby inhibiting tumor progression.

### D166 enhances sensitivity to anti-PD-1 therapy

**Figure 7:**
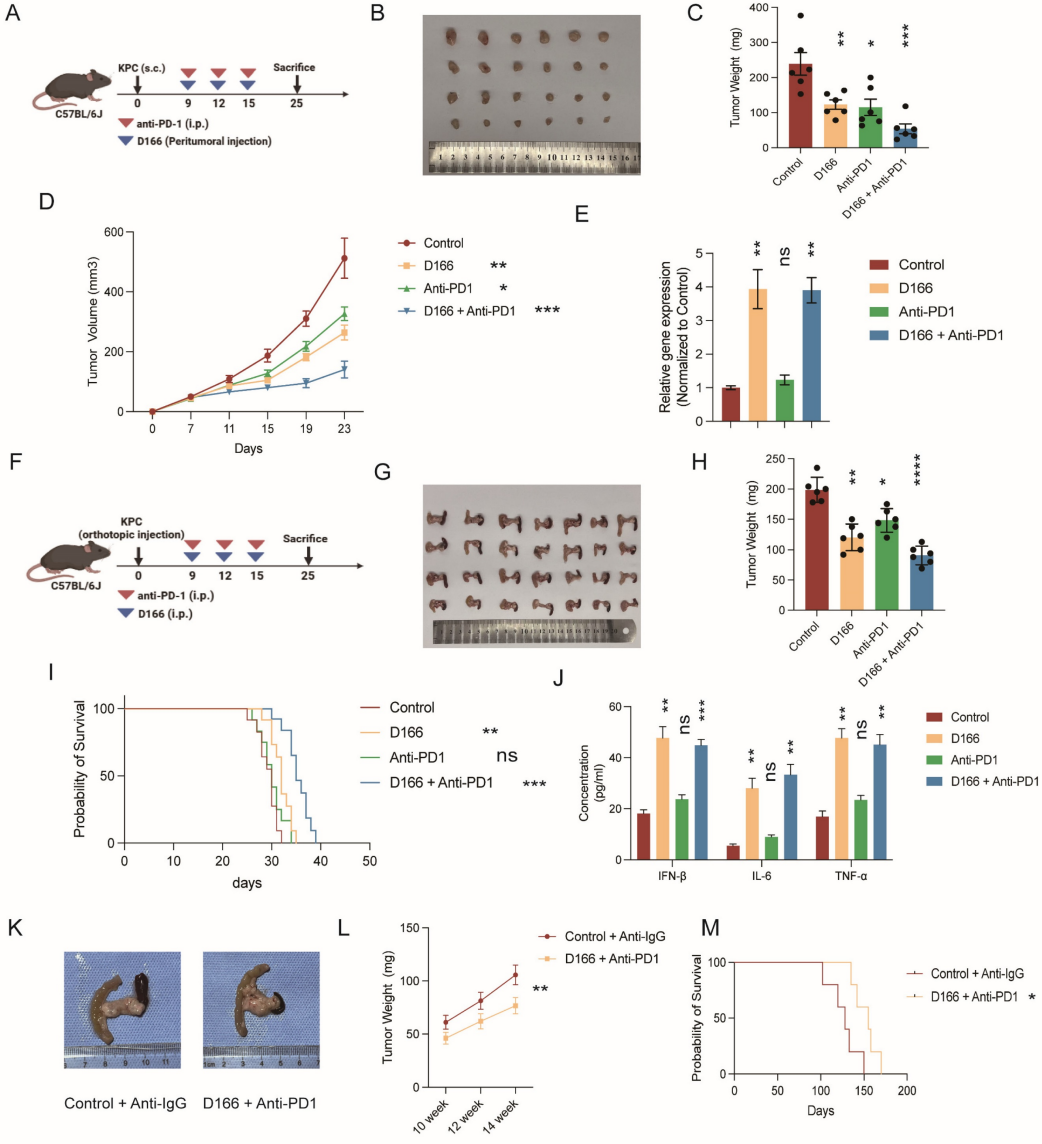
** D166 enhances of anti-PD-1 therapy sensitivity. (A)** Schematic diagram of mice subcutaneous pancreatic tumor model with D166 and anti-PD1 treatment. **(B-D)** Representative image (B), tumor weight (C) and growth curve (D) of mice subcutaneous pancreatic tumor model with D166 and anti-PD1 treatment (male, n=6). **(E)** Relative ISG gene expression in mice tumors above. **(F)** Schematic diagram of mice orthotopic pancreatic tumor model with D166 and anti-PD1 treatment. **(G-H)** Representative image (G) and tumor weight (H) of mice orthotopic pancreatic tumor model with D166 and anti-PD1 treatment. (male, n=6). **(I)** Survival cure of mice orthotopic pancreatic tumor model with D166 and anti-PD1 treatment. (n=10). **(J)** The concentration of IL-6, IFN-β and TNF-α in tumor microenvironment measured by ELISA. **(K)** Representative image of KPC mice treated with or without D166 and anti-PD1 antibody. **(L)** Tumor weight in KPC mice pancreas at 10, 12 and 14 weeks old treated with or without D166 and anti-PD1 antibody (n=3). **(M)** Survival curve of KPC mice treated with or without D166 and anti-PD1 antibody (n=5). Tumor weight and cytokine levels were compared using two-tailed unpaired Student's t-tests, while survival differences in Kaplan-Meier curves were analyzed using the log-rank test, calculating the statistical differences between each treatment group and the control group. *p < 0.05; **p < 0.01; ***p < 0.001; ****p < 0.0001.

As anti-PD-1 therapy exhibits limited efficacy in pancreatic cancer, we hypothesized that D166 could serve as a sensitizer for anti-PD-1 therapy. We combined D166 with an anti-PD-1 therapy and observed that the combination treatment markedly suppressed the progression of pancreatic cancer in the C57BL/6 subcutaneous tumor model (Figure 7A-D). PCR results showed a notably upregulated expression of ISGs, following D166 treatment (Figure 7E), indicating the activation of the cGAS-STING pathway. Similarly, in the orthotopic pancreatic tumor model, combination of D166 and anti-PD-1 significantly reduced tumor size and prolonged mouse survival (Figure 7F-I). ELISA further revealed substantial increases in IL-6, IFN-β, and TNF-α levels within the TME (Figure 7J).

Additionally, we used a KPC [C57BL/6-Krastm1(LSL-G12D)Trp53tm1(LSL-R172H)Tg(Pdx1-Cre)] transgenic mouse model, which spontaneously develops pancreatic cancer, and administered a combination of D166 and anti-PD-1. This combination therapy significantly slowed tumor progression and prolonged survival (Figure 7K-M). Therefore, the combination therapy involving D166 and anti-PD-1 represents a promising therapeutic approach for pancreatic cancer, where D166 functions as a STING agonist and serves as a sensitizer to anti-PD-1 therapy.

## Discussion

Pancreatic cancer exhibits high incidence, low survival rates, and limited treatment options, earning it the title “king of cancers.” To date, no available immunotherapy has proven effective against pancreatic cancer 25, and enhancing therapeutic efficacy and improving prognosis remain critical challenges. In this study, we introduced D166, a novel small-molecule STING agonist synthesized from MSA-2, that exhibits improved stability and therapeutic potency against pancreatic tumors. Our findings revealed that D166 activated the cGAS-STING pathway in a time- and dose-dependent manner in patient-derived pancreatic tumor organoid models. Furthermore, D166 monotherapy significantly inhibited pancreatic tumor progression and reshaped the immune microenvironment of pancreatic cancer. Moreover, a combination of D166 with anti-PD-1 therapy effectively suppressed tumor growth in mouse models and organoids, prolonging survival. These findings underscore promising new possibilities for pancreatic cancer treatment.

The highly immunosuppressive microenvironment of pancreatic cancer significantly contributes to its immunotherapy resistance 26,27. It contains high concentrations of immunosuppressive cells and cytokines that inhibit the tumor-killing functions of NK cells, T cells, and macrophages 28. Immune activators can play a crucial role in overcoming these barriers. D166 can activate both non-specific and specific immune cells, enhancing cytotoxicity and T cell proliferation, and promoting macrophage polarization toward the anti-tumor M1 phenotype. Notably, pancreatic tumors may become less rigid following D166 treatment, similar to breast and lung cancers, possibly due to reduced fibroblasts and collagen content. This structural change can potentially enhance immune cell infiltration and improve the efficacy of combination therapies such as anti-PD-1 antibody treatment.

Initially identified as a defense mechanism against viruses, the cGAS-STING pathway has recently been recognized for its critical role in anti-tumor immunity 5,13. The cGAS-STING pathway modulates anti-tumor immunity through multiple mechanisms. Upon activation, STING induces the release of type I IFNs, enhancing the proliferation and function of NK cells, thereby promoting tumor cell destruction 29. Additionally, STING activation can trigger the NLRP3 pathway, leading to direct tumor cell death 30. STING also promotes the M2 to M1 transition of macrophages in breast cancer 31. However, a study published in Nature reported that STING agonists may induce B cell differentiation into regulatory B cells, which secrete IL-35 to suppress NK cell proliferation, weakening their anti-tumor response 32. Therefore, the use of STING agonists as a monotherapy remains controversial and faces certain challenges.

The development of various STING agonists has garnered increasing attention with a growing understanding of the role of the cGAS-STING pathway in modulating anti-tumor immunity 33. Here, *in vitro* and *in vivo* studies demonstrated that D166 was more potent and stable in treating pancreatic cancer than most of the existing STING agonists, both in murine cell lines and human organoids, despite similar mechanisms of action. Several STING agonists have gradually entered clinical studies, and some have already failed. For instance, poor drug stability and systemic toxicity resulted in the termination of ADU-S100 and XMT-2056 in phase I clinical trials 34. Similarly, MK-1454 (Merck) demonstrated limited efficacy as a monotherapy, with no significant response in the single-agent treatment group 35. Nonetheless, several clinical trials investigating STING agonists are ongoing. For instance, 2321GCCC is being evaluated for the treatment of relapsed/refractory acute myeloid leukemia (NCT06626633), KL340399 for advanced solid tumors (NCT05549804), and CRD3874-SI for sarcoma and Merkel cell carcinoma (NCT06021626). Additionally, the combination of TAK-500 and pembrolizumab is being investigated for the treatment of locally advanced or metastatic solid tumors (NCT05070247). Nevertheless, a universally accepted strategy for maximizing the therapeutic potential of STING agonists in solid tumors remains unestablished.

Considering the current limitations of STING agonists, we selected C57BL/6 mice and patient-derived pancreatic cancer organoids to evaluate D166 efficacy. Traditionally, research on STING agonists has primarily been conducted using murine models 36. The C57BL/6 mouse strain, with fully functional innate and adaptive immune systems, serves as a suitable model for assessing the immunomodulatory effects of D166 as a STING agonist. In this study, we generated pancreatic cancer organoids from patient-derived tumor tissues, preserving the genetic and phenotypic heterogeneity of the original tumors. Compared with conventional two-dimensional tumor cell lines, organoid models more accurately recapitulated the tumor immune microenvironment, offering a more physiologically relevant system for predicting the clinical efficacy of D166^37^. Next, we established an organoid-immune cell co-culture system to further explore the effects of D166 on immune cell activation. Both *in vivo* and *in vitro* models in our study demonstrated that D166 effectively activated immune cells and exerted anti-tumor effects against pancreatic cancer. Given its efficacy in both murine models and human-derived organoids, D166 exhibits promising potential for clinical translation.

Although D166 has shown promising tumor-suppressive and immune-activating effects in preclinical models, certain limitations and potential off-target effects still persist. First, despite dual validation using C57 mice and patient-derived organoids, these models do not fully recapitulate the complex TME of human pancreatic cancer 38. In our *in vivo* models, D166 exerted its effects through direct tumor contact; however, efficient drug delivery remains a critical challenge 39. Pancreatic cancer is highly heterogeneous, and the role of the STING pathway may vary across different molecular subtypes. Consequently, whether D166 exerts a universal effect on pancreatic tumors or is only effective in specific subtypes warrants further investigation. Additionally, excessive activation of the STING pathway may trigger negative feedback regulatory mechanisms, leading to immune tolerance or adaptive resistance 19,40. Further studies are needed to evaluate the *in vivo* safety profile of D166, including its metabolic stability and pharmacokinetics. The potential off-target effects of STING agonists should also be explored, as hyperactivation of STING signaling may affect the function of normal immune cells. A transient but intense IFN response can lead to CD8+ T cell exhaustion, weakening anti-tumor immunity, whereas prolonged STING activation may impair the antigen-presenting capacity of dendritic cells 41. Furthermore, excessive STING activation is associated with cytokine storms, potentially leading to a systemic inflammatory response. Accordingly, future research should focus on optimizing the safe dosing regimen of D166 and assessing IFN-β-associated gene expression to mitigate unnecessary immune activation.

Our preclinical study presents a potential therapeutic avenue for pancreatic tumors, but still remains problems to be solved. Beyond understanding common adverse effects and resistance mechanisms associated with STING agonists, optimizing their pharmacokinetics and pharmacodynamics remains a key research focus 42. Despite its promising preclinical efficacy, a local injection of D166 is required for its therapeutic effects because of its low bioavailability following oral or intravenous administration, limiting its clinical feasibility. Future studies should explore structural modifications to enhance its half-life and enable targeted delivery. Chemotherapy remains the first-line treatment for pancreatic cancer. Beyond anti-PD-1 therapy, evaluating D166 in combination with other therapeutic modalities, such as radiotherapy or platinum-based chemotherapy, is also essential. Moreover, its potential synergy with other immune modulators, such as co-administration with TIGIT or LAG-3 inhibitors to alleviate T cell exhaustion or with IL-12 to enhance T cell infiltration into tumors, should be explored 43,44. Given the high heterogeneity of pancreatic cancer, further patient stratification is also essential. Well-defined biomarkers that can predict the sensitivity to STING agonists are currently unavailable. Future research should focus on identifying potential biomarkers of STING agonists, such as IFN levels, in the TME to predict patient responsiveness 45. Addressing these critical challenges is essential for STING agonists to advance toward clinical applications and offer hope to patients with cancer.

## Conclusion

In this study, we synthesized a novel and more stable small-molecule STING agonist, D166, and demonstrated its therapeutic efficacy for pancreatic cancer using both pancreatic tumor organoids and mouse models. D166 effectively remodels the tumor immune microenvironment by enhancing the cytotoxic function of T cells and promoting macrophage polarization towards the pro-inflammatory M1 phenotype. Furthermore, D166 acts as a sensitizer to anti-PD-1 therapy, improving the overall efficacy of immunotherapy in pancreatic cancer.

## Supplementary Material

Supplementary figures and tables.

## Figures and Tables

**Table 1:** D166 potently activates the STING signaling.

Activation folds of D166 against m- and h-STING		
**Compound**	**THP1-Dual Fold (10 μM)**	**Raw-lucia Fold (50 μM)**
**MSA-2**	43.09	22.59
**D166**	60.46	25.34
**D166 activates the expression of IFNβ and IL-6 in RAW-Lucia cells**		
**Compound**	**Relative expression** **(Ifnb/actin)**	**Relative expression** **(Cxcl10/actin)**
**CTRL**	1.00 ± 0.01	1.00 ± 0.08
**MSA-2**	125.70 ± 3.06	179.40 ± 2.58
**D166**	95.60 ± 3.69	158.00 ± 2.91

**Table 2:** Pharmacokinetic Parameters of MSA-2 and D166^a^

Compound	Route	T_1/2_ (h)	AUC_Inf_ (h*ng/mL)	Vz (L/kg)	CL (mL/min/kg)	MRT_Inf_ (h)	*F* (%)
**MSA-2**	i.v.	0.66	1372	0.70	12.3	0.26	
	p.o.	1.09	2852	-	-	0.89	69.3
**D166**	i.v.	0.75	750.1	1.45	22.5	0.26	
	p.o.	1.35	2120.1	-	-	1.10	93.8

^a^Values are the average of three runs. T_1/2_, half-life; AUC, area under the plasma concentration-time curve; Vz, volume of distribution; CL, clearance; MRT, mean residence time; *F*, oral bioavailability;

**Table 3:** Observed Metabolites of D166 in Mouse, Rat, Dog and Human Liver Microsomes after 60 min Incubation

Metabolite	Retention Time (min)	*m/z* (+)	Metabolic Pathway	Relative Peak Area Abundance (%)			
				MLM	RLM	DLM	HLM
M1	7.87	317.0960	Mono-oxygenation	0.1	0.1	D	D
M2	9.12	300.0616	Demethylation and mono-oxygenation	0.2	D	D	D
M3*	10.41	284.0667	Demethylation	5.5	2.2	1.4	1.5
M4	11.05	317.0960	Mono-oxygenation	D	D	D	D
P (D166)	12.25	301.1011	-	94.2	97.7	98.6	98.5

Note: P = parent; MLM, Mouse Liver Microsomes; RLM, Rat Liver Microsomes; DLM, Dog Liver Microsomes; HLM, Human Liver Microsomes; D = Detected; ND = not detected; M3* was detected in T= 0 min and standard samples, it was partly contributed from the impurity; the relative abundances of the parent and metabolites were calculated based on their selected ion chromatographic peak areas.

## Abbreviations

PDAC: Pancreatic ductal adenocarcinoma
STING: stimulator of interferon genes
CAF: cancer-associated fibroblasts
ECM: extracellular matrix
MDSC: myeloid-derived suppressor cells
Treg: regulatory T cells
PSC: pancreatic stellate cells
IFN: type I interferons
IFNAR: type I interferons alterative receptor
DMXAA: 5,6-Dimethylxanthenone-4-acetic acid
CDN: Cyclic dinucleotides
AO: Acridine orange
PI: propidium iodide
STR: short tandem repeat
M-CSF: Macrophage colony-stimulating factor
HE: hematoxylin and eosin
IHC: immunohistochemistry
IF: immunofluorescence
ELISA: enzyme-linked immunosorbent assay
ISG: interferon-stimulated gene
IL-6: interleukin 6
TNF-α: tumor necrosis factor alpha
