# Supplementary material for: Identification of a Novel Small Molecule STING Agonist Reshaping the Immunomicroenvironment of Pancreatic Ductal Adenocarcinoma
Source: Int J Biol Sci. 2025 May 27;21(8):3555–72. doi: 10.7150/ijbs.107837 (PMC12160547; doi:10.7150/ijbs.107837)
Supplement: Supplementary file 1 — Supplementary figures and tables. [file ijbsv21p3555s1.pdf]

Supplementary of

"Identification of a Novel Small Molecule STING Agonist Reshaping the Immunomicroenvironment of Pancreatic Ductal Adenocarcinoma"

Pengyi Liu[1,2,3, #], Minmin Shi[1,2,3, #], Yang Liu [1,2,3, #], Yihao Liu[1,2,3], Jiayu Lin[1,2,3], Shuyu Zhai[1,2,3], Yizhi Cao[1,2,3], Dongjie Chen[1,2,3], Yongsheng Jiang[1,2,3], Chenghong Peng[1,2,3], Lei Zhang[4, *], Chunyong Ding[5,6, *], Lingxi Jiang[1,2,3, *], Baiyong Shen[1,2,3, *]

1 Department of General Surgery, Pancreatic Disease Center, Ruijin Hospital, Shanghai Jiao Tong University School of Medicine, Shanghai 200025, China.

2 Research Institute of Pancreatic Diseases, Shanghai Key Laboratory of Translational Research for Pancreatic Neoplasms, Shanghai Jiao Tong University School of Medicine, Shanghai 200025, China.

3 State Key Laboratory of Oncogenes and Related Genes, Institute of Translational Medicine, Shanghai Jiao Tong University, Shanghai 200025, China.

4 Ruijin Department of General Surgery, Ruijin Hospital, School of Medicine, Shanghai Jiaotong University, Shanghai, China

5 Shanghai Frontiers Science Center for Drug Target Identification and Delivery, School of Pharmaceutical Sciences, Shanghai Jiao Tong University, Shanghai 200240, China.

6 National Key Laboratory of Innovative Immunotherapy, Shanghai Jiao Tong University, 800 Dongchuan Road, Minhang District, Shanghai 200240, China.

Supplementary Methods

1. Chemical synthesis of D166

D166 was chemically synthesized as illustrated in Figure 1A. All commercially available starting materials, reagents, and solvents were sourced from standard suppliers and used without additional purification. Proton nuclear magnetic resonance (1H NMR) spectra were recorded using a Varian Mercury 400 MHz NMR spectrometer. Chemical shifts (δ) are reported in parts per million (ppm) relative to an internal tetramethylsilane (TMS) standard, and coupling constants (J) are provided in Hertz (Hz). The purity of all synthesized compounds used for biological testing was confirmed to be greater than 95%.

1.1 benzo[b]thiophene-5,6-diol (2). A mixture of compound 1 (10.00 g, 51.48 mmol) and pyridine hydrochloride (17.85 g, 154.44mmol) was prepared in a sealed tube and stirred at 190 °C for 4 h. Once the reaction solution had cooled to room temperature, it was diluted with water and extracted with ethyl acetate for three times. The combined organic layer was washed with brine, dried over Na2SO4, and concentrated in vacuo. The residue was purified using a silica gel column to afford the desired compound 2 in quantitative yield. 1H NMR (400 MHz, DMSO-d6) δ 9.17 (s, 1H), 9.08 (s, 1H), 7.35 (d, J = 5.4 Hz, 1H), 7.23 (s, 1H), 7.16 (s, 1H), 7.16 – 7.14 (m, 1H).

1.2 5,6-bis(methoxy-d3)benzo[b]thiophene (3). To a solution of compound 2 (5.98 g, 51.47 mmol) in DMSO (80 mL), deuterated iodomethane (8.01 mL, 128.67 mmol) and potassium hydroxide (11.55 g, 205.87 mmol) were added. After stirring for 3.5 h at room temperature, the mixture was poured into the water and extracted with ethyl acetate. The combined organic layer was washed with brine, dried over Na2SO4, and concentrated in vacuo. The residue was purified by column chromatography to afford the desired compound 3 in 84% yield. 1H NMR (400 MHz, Chloroform-d) δ 7.30 (s, 1H), 7.28 (d, J = 5.4 Hz, 1H), 7.24 (s, 1H), 7.21 (d, J = 5.4 Hz, 1H).

1.3 4-(5,6-bis(methoxy-d3)benzo[b]thiophen-2-yl)-4-oxobutanoic acid (D166). A suspension of succinic anhydride (5.63 g, 56.23 mmol) in 1,2-dichloroethane (150 mL) was added aluminium chloride (10.00 g, 74.97 mmol) at 0 °C under an argon atmosphere. Subsequently, a solution of compound 3 (7.50 g, 37.49 mmol) in 1,2-dichloroethane (50 mL) was added dropwise over a period of 15 min. Following an additional 0.5 h at 0 °C, the reaction was then heated to 45 °C overnight until no further starting materials were detected by thin-layer chromatography (TLC). The mixture was poured into ice-water mixture and neutralised with 4N aqueous hydrochloric acid solution. After stirring for 0.5 h, the mixture was filtered. The crude filtering cake was then triturated with ethanol and filtered. The resulting solid was collected and dried, yielding the desired D166 in 48% yield. 1H NMR (400 MHz, DMSO-d6) δ 12.18 (s, 1H), 8.20 (s, 1H), 7.59 (s, 1H), 7.47 (s, 1H), 3.26 (t, J = 6.4 Hz, 2H), 2.60 (t, J = 6.3 Hz, 2H).

Supplementary Figures

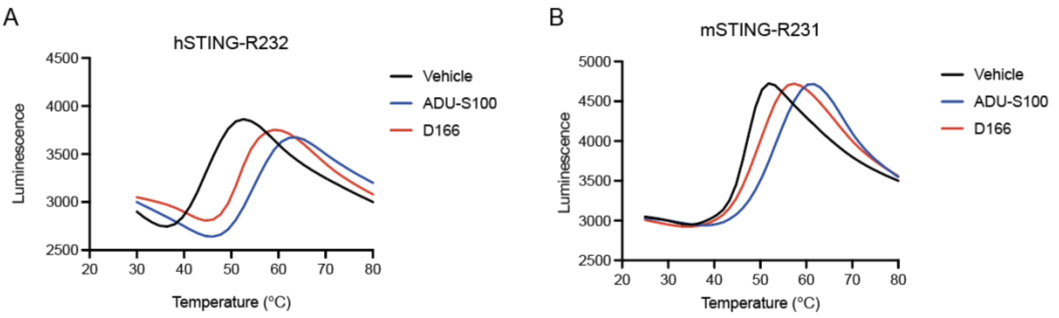

Figure S1 Thermal stability of D166. (A-B) D166 increased the thermal stability of human STING variants (R232) and mouse STING (R231) in differential scanning fluorimetry (DSF) assay. The data were shown as the mean value of fluorescence (n = 3).

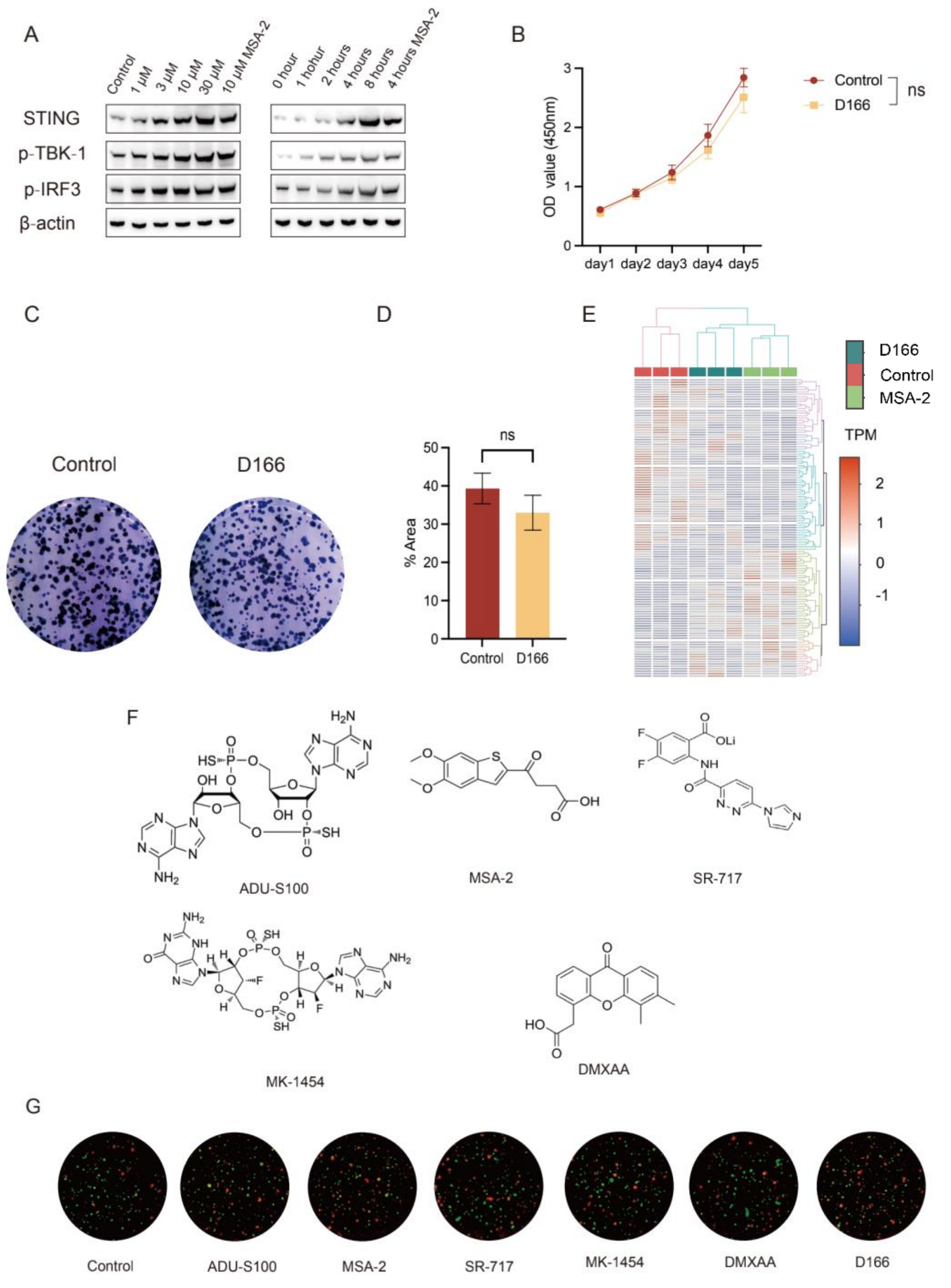

Figure S2 D166 activates STING pathway without affecting proliferation. (A) Western Blot showing cGAS-STING pathway protein expression of Panc-1 cell line when treated by different D166 concentration. (B) CCK8 of Panc-1 cell line when treated by 10μM D166. n=3. (C-D) Representative image (C) and statistic (D) of Colony assay of Panc-1 cell line when treated by 10μM D166. (E) Global gene

expression after adding 10μM D166 or MSA-2, compared with control group. (F) Structure of STING agonists ADU-S100, MSA-2, SR-717, MK-1454 and DMXAA. (G) Representative organoid image after different STING agonists treatment and AO/PI staining. $^{*}p < 0.05$; $^{**}p < 0.01$; $^{***}p < 0.001$; $^{****}p < 0.0001$.

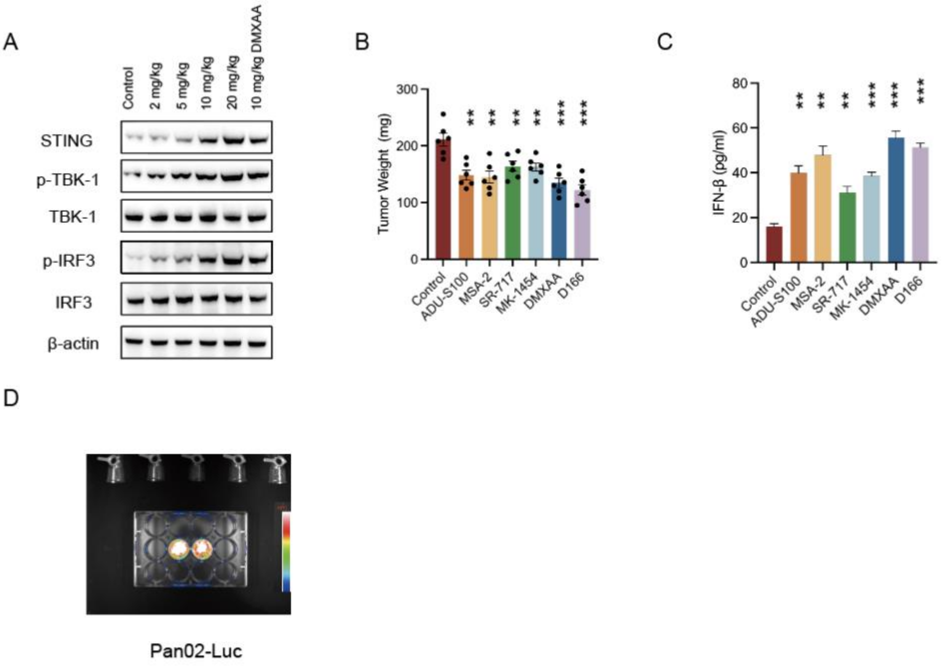

Figure S3 D166 monotherapy inhibits orthotopic PDAC progression. (A) Western Blot showing cGAS-STING pathway protein expression in pancreatic orthotopic tumor model. (B-C) Tumor weight (B) and IFN-β level (C) of mice orthotopic PDAC after treatment by different STING agonists. (D) In vivo imaging of Pan02-Luc cell line treated with 150 μg/ml D-Luciferin by Tanon ABL-X5. *$p < 0.05$; **$p < 0.01$; ***$p < 0.001$; ****$p < 0.0001$.

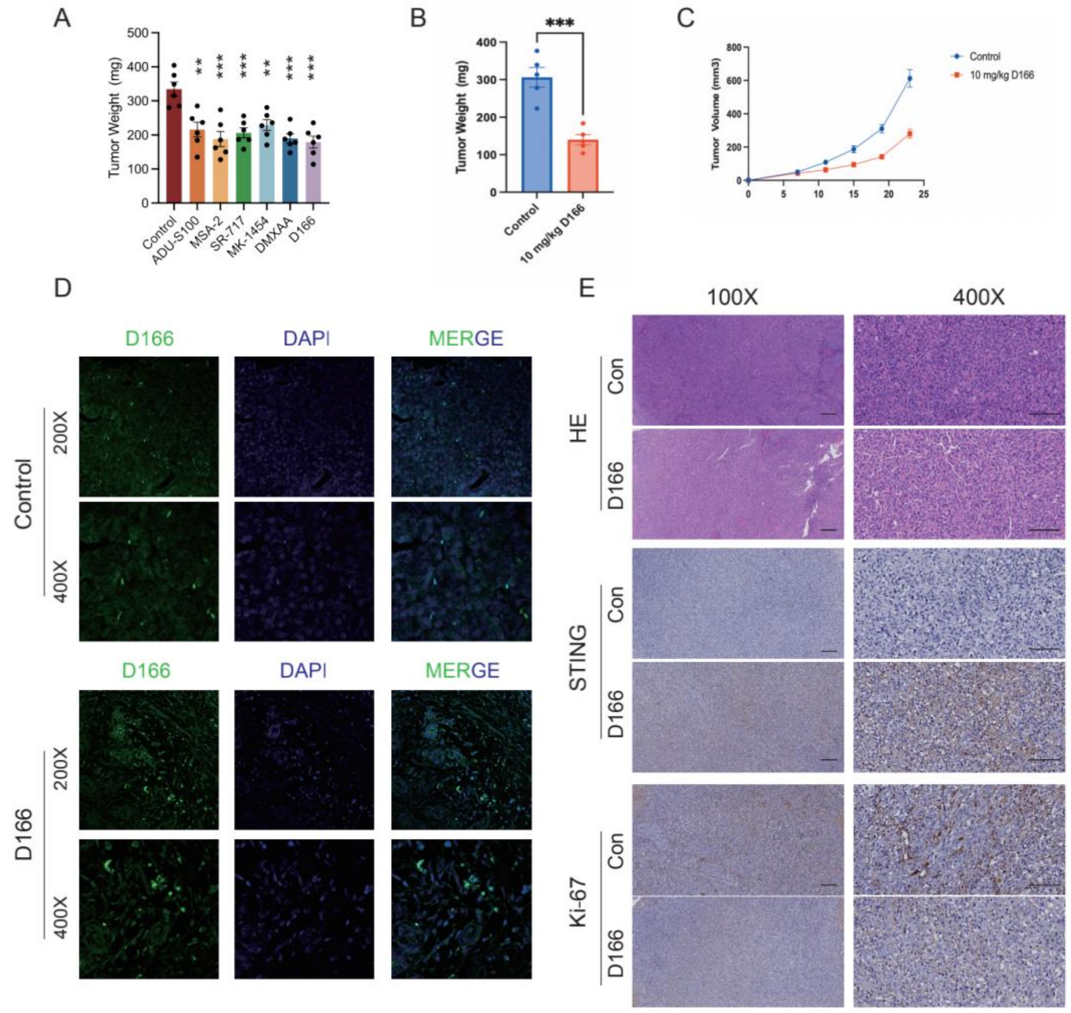

Figure S4 D166 monotherapy inhibits subcutaneous PDAC progression. (A) Tumor weight of mice subcutaneous PDAC after treatment by different STING agonists. (B-C) Tumor weight (B) and growth curve (C) of mice subcutaneous pancreatic tumor mono-treated by 10mg/Kg D166. (D-E) IF (D) and IHC (E) images of subcutaneous pancreatic tumor model after treated by 10 mg/Kg D166. Scale bar = 50 μm. *$p < 0.05$; **$p < 0.01$; ***$p < 0.001$; ****$p < 0.0001$.

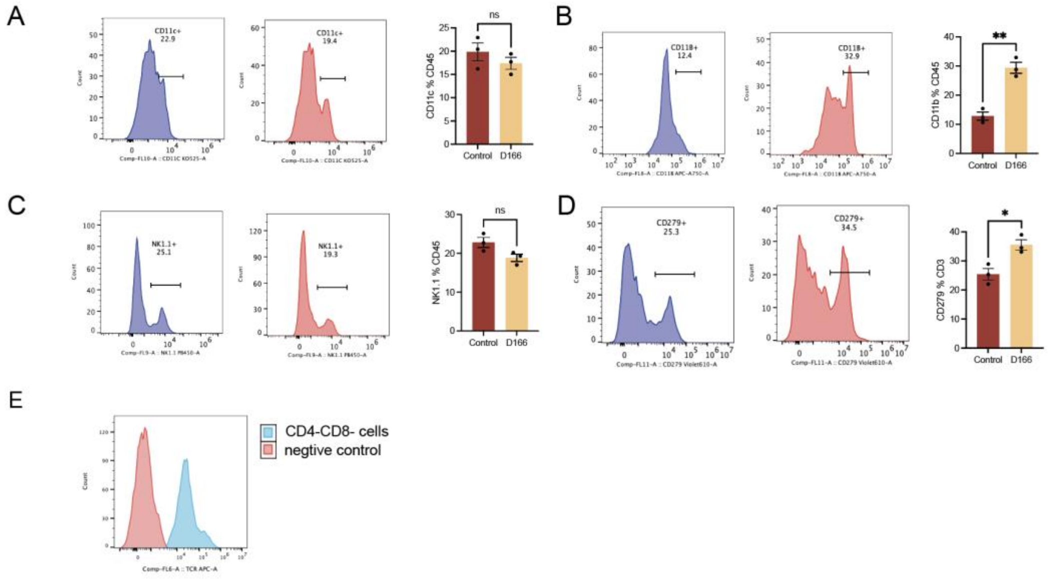

Figure S5 D166 Reshapes the Tumor Immune Microenvironment in Pancreatic Cancer. (A-C) Percentage of CD11c (A), CD11b (B) and NK1.1 (C) expression and statistical analysis in CD45+ cells from pancreatic orthotopic tumor by flow cytometry. (D) CD279 (PD-1) expression and statistical analysis in CD3+ T cells from pancreatic orthotopic tumor by flow cytometry. (E) TCRγδ expression in CD3+CD4-CD8- cells from pancreatic orthotopic tumor by flow cytometry. *$p < 0.05$; **$p < 0.01$; ***$p < 0.001$; ****$p < 0.0001$.

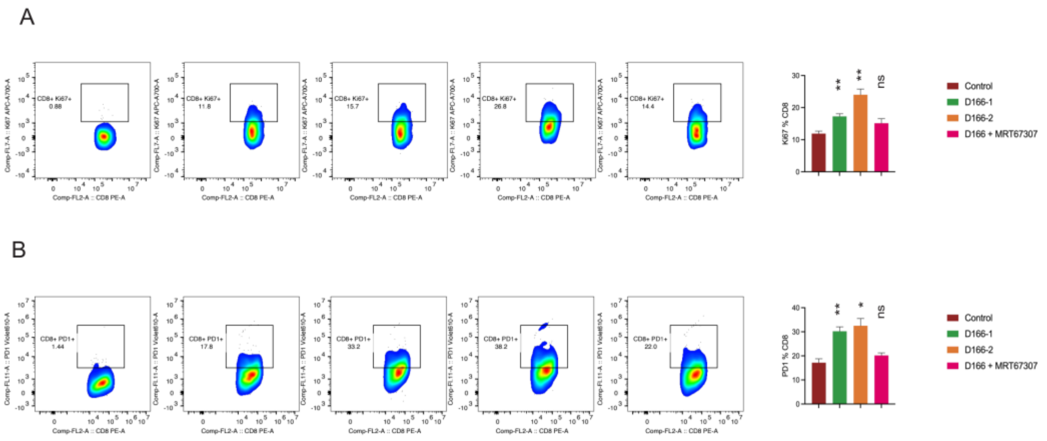

Figure S6 D166 can activate T cells co-cultured with organoids. (A-B) The expression of Ki67 (A) and PD1 (B) in co-cultured T cells by flow. The representative flow cytometry plots are shown on the left, and the statistical analysis is shown on the right. *p < 0.05; **p < 0.01; ***p < 0.001; ****p < 0.0001.

Table S1 Antibodies used in this article

Antibodies for Western Blot			
**Antibody**	**Company**	**Cat. No.**	**Species**
Mouse-Reactive STING Pathway Antibody Sampler Kit	Cell Signaling Technology	#16029	Rabbit
Human-Reactive STING Pathway Antibody Sampler Kit	Cell Signaling Technology	#38866	Rabbit
NF-κB p65 (D14E12)	Cell Signaling Technology	#8242	Rabbit
Phospho-NF-κB p65 (Ser536)	Cell Signaling Technology	#3033	Rabbit
β-Actin (13E5) Rabbit mAb	Cell Signaling Technology	#4970	Rabbit
IFN-beta Rabbit pAb	ABclonal	A23651	Rabbit
Ki67 Rabbit mAb	ABclonal	A20018	Rabbit

Antibodies for flow cytometry			
**Antibody**	**Company**	**Cat. No.**	**Species**
BD Horizon™ APC-R700 Rat Anti-Mouse CD45	BD Pharmingen	565478	Mouse
BD Pharmingen™ PerCP-Cy™5.5 Hamster Anti-Mouse CD3e	BD Pharmingen	551163	Mouse
BD Pharmingen™ Alexa Fluor® 488 Rat Anti-Mouse CD4	BD Pharmingen	557667	Mouse
BD Pharmingen™ PE Rat Anti-Mouse CD8a	BD Pharmingen	553032	Mouse
BD Horizon™ BV510 Hamster Anti-Mouse CD11c	BD Pharmingen	562949	Mouse
BD Horizon™ PE-CF594 Rat Anti-Mouse F4/80	BD Pharmingen	565613	Mouse
BD Horizon™ BV605 Hamster Anti-Mouse CD279 (PD-1)	BD Pharmingen	563059	Mouse
CD11b Monoclonal Antibody (ICRF44), APC-eFluor™ 780	ThermoFisher	47-0118-42	Mouse
Brilliant Violet 421™ anti-mouse CD206 (MMR) Antibody	Biolegend	141717	Mouse

BD Horizon™ BV421 Mouse Anti-Mouse NK-1.1	BD Pharmingen	562921	Mouse
TCR gamma/delta Monoclonal Antibody, APC	ThermoFisher	17-5711-82	Mouse
PE anti-human CD8 Antibody	Biolegend	344706	Human
Brilliant Violet 605™ anti-human CD279 (PD-1) Antibody	Biolegend	329923	Human
Pacific Blue™ anti-human/mouse Granzyme B Recombinant Antibody	Biolegend	372217	Human
BD Pharmingen™ APC Mouse Anti-Human IFN-γ	BD Pharmingen	562017	Human
Alexa Fluor® 700 anti-human Ki-67 Antibody	Biolegend	350529	Human
FITC anti-human CD68 Antibody	Biolegend	333806	Human
PE/Cyanine7 anti-human CD80 Antibody	Biolegend	375407	Human
Brilliant Violet 421™ anti-human CD86 Antibody	Biolegend	381005	Human
Brilliant Violet 421™ anti-human CD163 Antibody	Biolegend	333611	Human
APC anti-human CD206 (MMR) Antibody	Biolegend	321109	Human
BD Horizon™ Fixable Viability Stain 440UV	BD Pharmingen	566332	Mouse/Human
Transcription Factor Buffer Set 100Tst	BD Pharmingen	562574	Mouse/Human
Cellular stimulation cocktail eBioscience™ (500 X)	ThermoFisher	00-4970-93	Mouse/Human

Table S2 PCR primer used in this article

Primers for RNA quantitation		
Gene Symbol	Forward Sequence	Reverse Sequence
ACTB	GTCATTCCAAATATGAGATGCGT	GCTATCACCTCCCCTGTGTG
IFNG	TCGGTAACTGACTTGAATGTCCA	TCGCTTCCCTGTTTTAGCTGC
GZMB	CCCTGGGAAAACACTCACACA	GCACAACTCAATGGTACTGTCG
PDCD1	CCAGGATGGTTCTTAGACTCCC	TTTAGCACGAAGCTCTCCGAT
CD86	CTGCTCATCTATACACGGTTACC	GGAAACGTCGTACAGTTCTGTG
NOS2	ACATCGACCCGTCCACAGTAT	CAGAGGGGTAGGCTTGTCTC
CD163	GACGCATTTGGATGGATCATGT	CCCACCGTCCTTGGAATTTGA
ARG1	TGGACAGACTAGGAATTGGCA	CCAGTCCGTCAACATCAAAACT

Table S3 ELISA kit used in this article

ELISA kit	Company	Cat. No.	Reactivity
Mouse IL-6 ELISA Kit	ABclonal	RK00008	Mouse
Mouse IFN beta ELISA Kit	ThermoFisher	424001	Mouse
Mouse TNF-alpha ELISA Kit	ABclonal	RK00027	Mouse
Human IL-6 ELISA Kit	ABclonal	RK00004	Human
Human Interferon Beta ELISA Kit (IFNb)	ABclonal	RK01630	Human
Rabbit anti-Human IFN-gamma mAb	ABclonal	RMK0039	Human
Human TNF-alpha ELISA Kit	ABclonal	RK00030	Human

Table S4 Key reagents used in this article

Product	Company	Cat. No.
MRT67307 (TBK-1 inhibitor)	MedChemExpress	HY-13018
ADU-S100	MedChemExpress	HY-12885
MSA-2	MedChemExpress	HY-136927
SR-717	MedChemExpress	HY-131454
Ulevostinag (MK-1454)	MedChemExpress	HY-139586
Vadimezan (DMXAA)	MedChemExpress	HY-10964
Corning Matrigel Matrix	Corning	356234
InVivoMAb anti-mouse PD-1 (CD279)	BioXCell	BE0146
InVivoMAb mouse IgG1 isotype control	BioXCell	BE0083
EasySep™ Human CD8+ T Cell Isolation Kit	Stemcell	17953
EasySep™ Human Monocyte Isolation Kit	Stemcell	19359
Human CD3/CD28 T Cell Activation Beads	Biolegend	422604
Recombinant Human M-CSF	Biolegend	574804
